# One mechanism of spleen-kidney yang deficiency IBS-D: intestinal microbiota affect ATPase

**DOI:** 10.3389/fmicb.2025.1595418

**Published:** 2025-08-12

**Authors:** Qi Long, Liwen Li, Na Deng, Zhoujin Tan

**Affiliations:** 1 School of Traditional Chinese Medicine, Hunan University of Chinese Medicine, Changsha, China; 2 Hunan Key Laboratory of Traditional Chinese Medicine Prescription and Syndromes Translational Medicine, Changsha, China

**Keywords:** intestinal microbiota, ATPase, spleen-kidney yang deficiency IBS-D, energy metabolism, intestinal digestive enzymes

## Abstract

**Background:**

This study aimed to investigate the effects of adenine and *Folium senna* combined with restraint tail-clamping stress method on the intestinal microbiota of mice with spleen-kidney yang deficiency type diarrhea predominant irritable bowel syndrome (IBS-D) from the perspective of energy metabolism.

**Methods:**

Twenty SPF-grade female mice were randomly divided into two groups, normal group (CN group) and model group (MD group), with 10 mice per group. A spleen-kidney yang deficiency IBS-D model was established using adenine and *Folium senna* combined with restraint tail-clamping stress. After model establishment, enzyme-linked immunosorbent assay (ELISA) was used to detect the activity of Na^+^-K^+^-ATPase and Ca^2+^-Mg^2+^-ATPase; spectrophotometry was used to measure intestinal digestive enzyme activity; fluorometric diacetate (FDA) hydrolysis spectrophotometry was used to detect intestinal microbial activity; and 16S rRNA sequencing was performed to identify intestinal microbiota in the intestinal contents.

**Results:**

(1) ELISA showed a significant decrease in Na^+^-K^+^-ATPase and Ca^2+^-Mg^2+^-ATPase activity in spleen-kidney yang deficiency IBS-D model mice. (2) Intestinal enzyme activity analysis revealed increased lactase and amylase activity, and decreased sucrase activity in model mice. (3) Intestinal microbial activity analysis demonstrated significantly enhanced in microbial activity in spleen-kidney yang deficiency IBS-D model mice. (4) Correlation analysis revealed a significant positive correlation between *Heminiphilus, Clostridium, Desulfovibrio* and Ca^2+^-Mg^2+^-ATPase, a significant positive correlation between *Heminiphilus* and Na^+^-K^+^-ATPase, a significant positive correlation between *Pediococcus* and sucrase, and a significant negative correlation between *Clostridium* and sucrase.

**Conclusion:**

Reduced Na^+^-K^+^-ATPase and Ca^2^^+^-Mg^2^^+^-ATPase activities, increased sucrase and amylase activities, decreased lactase activity, and enhanced intestinal microbial activity may constitute important mechanisms underlying the development of spleen-kidney yang deficiency IBS-D.

## 1 Introduction

Irritable Bowel Syndrome (IBS) is a prevalent functional gastrointestinal condition, typically defined by recurring stomach pain and irregular bowel movements or alterations in bowel habits (Mearin et al., 2016). Diarrhea-predominant irritable bowel syndrome (IBS-D) is the most prevalent subtype (Pan et al., 2022; Long et al., 2017). Modern medicine currently regards the etiology of IBS-D as multifactorial, encompassing heightened visceral sensitivity, aberrant gastrointestinal motility, dysbiosis, intestinal inflammation, dysregulation of the brain-gut axis, and psychological influences (Kassam et al., 2013; Lian et al., 2021). These factors collectively influence the intricate pathophysiology of the illness. In Traditional Chinese Medicine (TCM), the clinical manifestation of IBS-D is classified as “diarrhea” and “abdominal pain”(Kang et al., 2020), and it is further delineated into five fundamental syndromes: liver stagnation with spleen deficiency, spleen deficiency with dampness, kidney yang deficiency with spleen deficiency, spleen-stomach damp-heat, and mixed cold-heat syndrome (Gastrointestinal functional diseases cooperative group, Chinese society of gastroenterology, Chinese medical association, gastrointestinal dynamics group, Chinese society of gastroenterology, 2020). The syndrome of spleen-kidney yang deficiency is common, often manifesting with symptoms like watery stools, undigested food, abdominal pain relieved by warmth, and lumbar soreness with cold extremities. The pathophysiology of IBS-D remains unclear, and the syndrome is characterized by repeated episodes and medical challenges; thus, additional study is essential to elucidate its underlying mechanisms.

Recent studies indicate a strong correlation between intestinal microbiota and IBS-D. Dysbiosis may facilitate the development of IBS-D by altering the intestinal barrier, triggering immunological responses, and heightening visceral hypersensitivity (Bai and Liu, 2023). Intestinal dysbiosis significantly contributes to the pathogenesis of IBS-D by altering microbial composition and diminishing the synthesis of metabolic byproducts, including short-chain fatty acids (SCFAs). Dysbiosis diminishes intestine colonization resistance (Zhou and Lu, 2013). The imbalance primarily occurs in the colon and small intestine, characterized by a reduction in helpful bacteria (e.g., *Bifidobacterium, Lactobacillus*) and an increase in pathogenic bacteria (e.g., *Escherichia coli*) in the colon, alongside a rise in microbiota abundance in the small intestine (Pittayanon et al., 2019). Dysbiosis can diminish the prevalence of short-chain fatty acid (SCFA)-producing bacteria, such as butyrate-producing bacteria, resulting in reduced SCFA levels, particularly butyrate, which subsequently impacts intestinal energy metabolism (Hu et al., 2020). The intestinal microbiome influences energy metabolism by regulating mitochondrial activity, hormone release, and metabolic byproducts (Ma et al., 2025). Butyrate, a primary energy source for colonic epithelial cells, modulates mitochondrial metabolism via many mechanisms, hence controlling mitochondrial function in colon cells (Zhang et al., 2018, 2022). Propionate, acetate, and butyrate participate in the tricarboxylic acid (TCA) cycle, enhancing ATP production to supply adequate energy for the body (Tang and Li, 2021). TNF-α, IL-1 and IL-6, play a significant role in promoting inflammatory responses (Hou et al., 2020). In the state of spleen-kidney yang deficiency, both IL-10 and TNF-α inflammatory factors are elevated, leading to inflammatory manifestations. Previous studies have shown that SCFAs can activate the nuclear factor kappa-B (NF-κB) pathway in intestinal epithelial cells, increasing TNF-α secretion and reducing IL-10 secretion (Bolte et al., 2020). SCFA may influence visceral hypersensitivity and alleviate inflammation by regulating gastrointestinal motility and the secretion of inflammatory factors (Deng et al., 2025). Additionally, studies have shown that gallic acid (GA) can effectively correct intestinal microbiota imbalance, increase SCFA levels, inhibit inflammation, and enhance tight junction protein expression, thus improving the intestinal barrier (Tian et al., 2025).

Na^+^-K^+^-ATPase and Ca^2^^+^-Mg^2^^+^-ATPase, the principal ATPases in the body, regulate ion concentrations and biological membrane functionality, acting as crucial markers of energy metabolism (Tremellen and Pearce, 2012). Na^+^-K^+^-ATPase, known as the sodium pump, situated in the mitochondrial inner membrane and cell membrane, modulates mitochondrial osmotic pressure and shape, and is crucial for energy conversion, signal transduction, and substance transport. Its function is directly associated with cellular resilience to injury and the efficiency of energy production (Simão et al., 2011). The Ca^2^^+^-Mg^2^^+^-ATPase modulates the calcium ion equilibrium between the cytoplasm and the sarcoplasmic reticulum (Liu et al., 2022). Impaired energy metabolism suppresses the activity of Na^+^-K^+^-ATPase and Ca^2^^+^-Mg^2^^+^-ATPase, resulting in intracellular buildup of Na^+^ and Ca^2^^+^, which exacerbates malfunction in energy synthesis and induces cellular damage (Pongkorpsakol et al., 2017). This metabolic disease can hinder water absorption in the gastrointestinal tract, resulting in diarrhea. We postulate that intestinal microbiota may affect energy metabolism by modulating ATPase activity, hence contributing to the pathophysiology of spleen-kidney yang deficiency IBS-D.

This research recreated the previous team's model of spleen-kidney yang deficiency IBS-D by observing mouse behavior, measuring microbial activity, and assessing digestive enzyme activity. The functions of Na^+^-K^+^-ATPase and Ca^2^^+^-Mg^2^^+^-ATPase were evaluated, and the microbial population within the small intestine contents was examined. The interplay of the small intestinal microbiota, energy metabolism, and spleen-kidney yang deficiency IBS-D was investigated.

## 2 Materials and methods

### 2.1 Materials

#### 2.1.1 Animals and feeding

Twenty female SPF-grade Kunming mice (18–22 g) were purchased from Hunan Slack Jingda Laboratory Animal Co., Ltd. (animal license number: SCXK (Xiang) 2019–0004). To eliminate the influence of sex on the intestinal microbiota of mice, this study only used female mice (Wu et al., 2022).

#### 2.1.2 Ethical approval

All animal experiments were approved by the Animal Ethics and Welfare Committee of Hunan University of Chinese Medicine, with ethical approval number HNUCM21-2404-26. The feed was provided by Beijing HFK Bioscience Co., Ltd. [Feed License: (2019) 06076].

#### 2.1.3 Experimental drugs and reagents

*Folium Senna* (Anhui Shenghaitang Traditional Chinese Medicine Co., Ltd., Batch No. 2019060561) at a concentration of 1 g/mL. adenine [Changsha Yaer Biology Co., LTD, Changsha, China, EZ7890C450(Wu et al., 2021), ortho-nitrophenyl β-D-galactopyranoside (ONPG, Shanghai Yuanye Bio-Technology Co., Ltd.], 3,5-dinitrosalicylic acid (DNS, Shanghai Runchen Bio-Technology Co., Ltd.), fluorescein diacetate (FDA, Shanghai Yuanye Bio-Technology Co., Ltd.), and acetone (Hunan Huihong Reagent Co., Ltd.) were used, The Na^+^-K^+^-ATPase kit (Lot JM-11845M2) and Ca^2+^-Mg^2+^-ATPase kit (Lot JM-12156M2) were both purchased from Jiangsu Jingmei Bio-Tech Co., Ltd.

### 2.2 Methods

#### 2.2.1 Animal grouping and modeling

After 3 days of adaptive feeding, 20 female mice were randomly assigned to normal and model group with 10 mice in each group. The IBS-D mouse model with spleen-kidney yang deficiency was induced by the administration of adenine and *Folium sennae* decoction combined with restraint-tail clamping stress on the basis of a previous study from our research team. In the week prior to modeling, the model group was gavaged with an adenine suspension (50 mg/(kg·d), 0.4 mL per mouse, once a day, for 14 consecutive days). Starting from the 8th day of modeling, the limbs of model group mice were restrained via a centrifuge tube, and the distal 1/3 of their tail was clamped with a long tail clamp. Both the restraint and intermittent tail-clamping durations were 1 h, which lasted for 7 consecutive days. Starting from day 10, model group mice were gavaged with *Folium sennae* decoction in the afternoon (10 g/(kg·d), 0.4 mL/mouse, once a day, for 5 consecutive days), whereas normal group received sterile water at the same frequency and volume (Deng et al., 2024).

#### 2.2.2 Model evaluation criteria

On the basis of the diagnostic criteria outlined in the “2023 Expert Consensus on TCM Diagnosis and Treatment of Diarrhea” and the “2017 Expert Consensus on TCM Diagnosis and Treatment of Irritable Bowel Syndrome,” we established assessment indices for spleen-kidney yang deficiencyIBS-D. Clinically, spleen-kidney yang deficiency IBS-D is defined by recurrent abdominal pain, bloating, and discomfort (primary symptoms), accompanied by cold intolerance, a preference for warmth/pressure, and lumbar/knee weakness (secondary symptoms), with diagnosis contingent upon the presence of both primary and secondary symptoms. We correlated clinical manifestations with quantifiable parameters by observing general behavior (activity level and lethargy) to indicate “fatigue and sluggishness”; measuring fecal water content to evaluate “dawn diarrhea with undigested food”; monitoring anal temperature, huddling, and arched-back behavior to correspond with “cold limbs,” and assessing changes in body weight, food intake, and water intake to signify “poor appetite and emaciation.”

#### 2.2.3 General characteristics observation

Observing the general condition of each group of mice before and after modeling, including body weight, food intake, water consumption, activity level, and mental state.

#### 2.2.4 Measurement of fecal moisture content

Simultaneously, fresh feces were collected from each group before modeling and at the initial stage of *Folium senna* gavage (day 8) to calculate the fecal moisture content. Fresh feces were dried to a constant weight and weighed to record the dry weight to calculate the fecal moisture content. Fecal moisture content (%) = (wet weight—dry weight)/wet weight × 100% (Qiao et al., 2023b).

#### 2.2.5 16S rRNA high-throughput sequencing of small intestinal contents microbiota

Intestinal content samples were collected on a sterile workbench to extract total DNA. The following primers were designed on the basis of conserved regions: forward primer 338F5′-ACTCCTACGGGAGGCAGCA3′ and the reverse primer 806R 5′-GGACTACHV GGGTWTCTAAT3′. Sequencing adapters were added to the ends of the primers, and the V3V4a hypervariable region of the 16S rRNA gene was selected for PCR amplification. The amplified PCR products were detected via 1.8% agarose gel electrophoresis, and the DNA concentration and purity were measured with a NanoDrop2000 (Thermo Scientific, USA). The PCR products were subsequently purified, quantified, and normalized to form a sequencing library (Qiao et al., 2023a).

#### 2.2.6 Collection of organs and calculation of organ indices

The complete spleen and thymus were collected. The organ index is calculated as follows: Organ index = Organ weight (g)/Body weight (g) × 100% (Qiao et al., 2023b).

#### 2.2.7 Open field test in mice

The open field test (OFT) is a method used to evaluate the spontaneous behavior, exploratory behavior (Sun et al., 2018). On the 14th day of modeling, the mice were placed in the testing room for an adaptation period of 30 min. Using a random number table method, 5 mice from each group were selected for testing in the KSYY-OP-V4.0 mouse open field real-time detection and analysis system. The movement distance and average speed of each mouse were observed over a 5-min period. After each mouse's experiment, the feces and urine were removed, and the testing box was wiped with 75% ethanol, allowing the ethanol to evaporate before this process was repeated 5 times to obtain an average value (Qiao et al., 2023a,b).

#### 2.2.8 Measurement of intestinal enzyme activity

After collecting the small intestinal contents under a laminar flow cabinet, each group of samples was placed in a sterile centrifuge tube containing an appropriate amount of sterile water and 7–8 glass beads, with a ratio of 3 g of contents to 50 ml of sterile water. The samples were vortexed for 2 min to ensure the complete release of enzyme-related substances. Then, the mixture was centrifuged at 3,000 rpm at 4°C for 10 min, and the supernatant was collected as the crude enzyme solution. To ensure the accuracy and comparability of the experiment, one blank control tube and three sample tubes were set up for each experiment. The activity of amylase was measured via the DNS colorimetric method. The activity of sucrase was determined at a wavelength of 540 nm, and the activity of lactase was measured via the ONPG method at a wavelength of 420 nm (Wu et al., 2021).

#### 2.2.9 Measurement of intestinal microbial activity

Samples of murine intestinal contents were collected and diluted with sterile water at a ratio of 3 g to 50 mL. For control group, FDA and acetone were added at a 1:200 ratio, while the sample group received only FDA. The mixtures were shaken for 90 min, then centrifuged at 2,000 r/min for 10 min to collect the supernatant. In the sample group, acetone was added to stop the reaction. Microbial activity per unit mass was measured at 490 nm (Wu et al., 2021).

#### 2.2.10 Measurement of Na^+^-K^+^-ATPase and Ca^2+^-Mg^2+^-ATPase in serum

The activities of Na^+^-K^+^-ATPase and Ca^2+^-Mg^2+^-ATPase were analyzed via ELISA. Whole blood samples from the mice were collected and centrifuged at 3,000 r/min for 10 min to separate the serum. The plate layout, sample addition, enzyme addition, incubation, washing, color development, and reaction termination were performed according to the guidelines provided in the ELISA kit. The OD value was measured at a wavelength of 450 nm via a microplate reader (Zhu et al., 2022).

#### 2.2.11 Bioinformatics analysis

(1) Use the QIIME2 table to compute the ASV-level α diversity index, producing ASV-level ranked abundance curves to assess the richness and evenness of ASVs across samples (Callahan et al., 2016).

(2) Use the Bray-Curtis metric (Bray and Curtis, 1957) for β-diversity analysis to examine the structural alterations of microbial communities in samples, and represent them using Principal Coordinate Analysis (PCoA) and Non-metric Multidimensional Scaling (NMDS).

(3) Utilize the R package “Venn Diagram” to create Venn diagrams that illustrate the shared and distinct ASVs among samples or groups. Execute LEfSe (Linear Discriminant Analysis Effect Size) with default parameters to identify differentially abundant taxa across groups.

(4) The PICRUSt2 tool predicts the functional abundance of samples in the KEGG database and filters for highly enriched metabolic pathways between groups.

(5) Employ Spearman analysis to investigate the association among digestive enzyme activity, microbial activity, Na^+^-K^+^-ATP-ase and Ca^2+^-Mg^2+^-ATP-ase activity in relation to the intestinal microbiome composition.

(6) Employing redundancy analysis (RDA) to investigate the interplay between the distinctive microbiota of intestinal contents and the functions of Na^+^-K^+^-ATP-ase and Ca^2+^-Mg^2+^-ATP-ase.

#### 2.2.12 Statistical methods

Using SPSS version 25.0 for statistical analysis. All measured data are presented as “mean ± standard deviation.” A *t*-test with independent samples is used for comparison between two groups if the data follows to a normal distribution. In the absence of a normal distribution, the Mann-Whitney U test is employed for comparisons between two groups. A significance level of α = 0.05 was set for all statistical tests.

## 3 Results

### 3.1 Effects of spleen-kidney yang deficiency IBS-D on the general behavioral observations in mice

**Figure 1:**
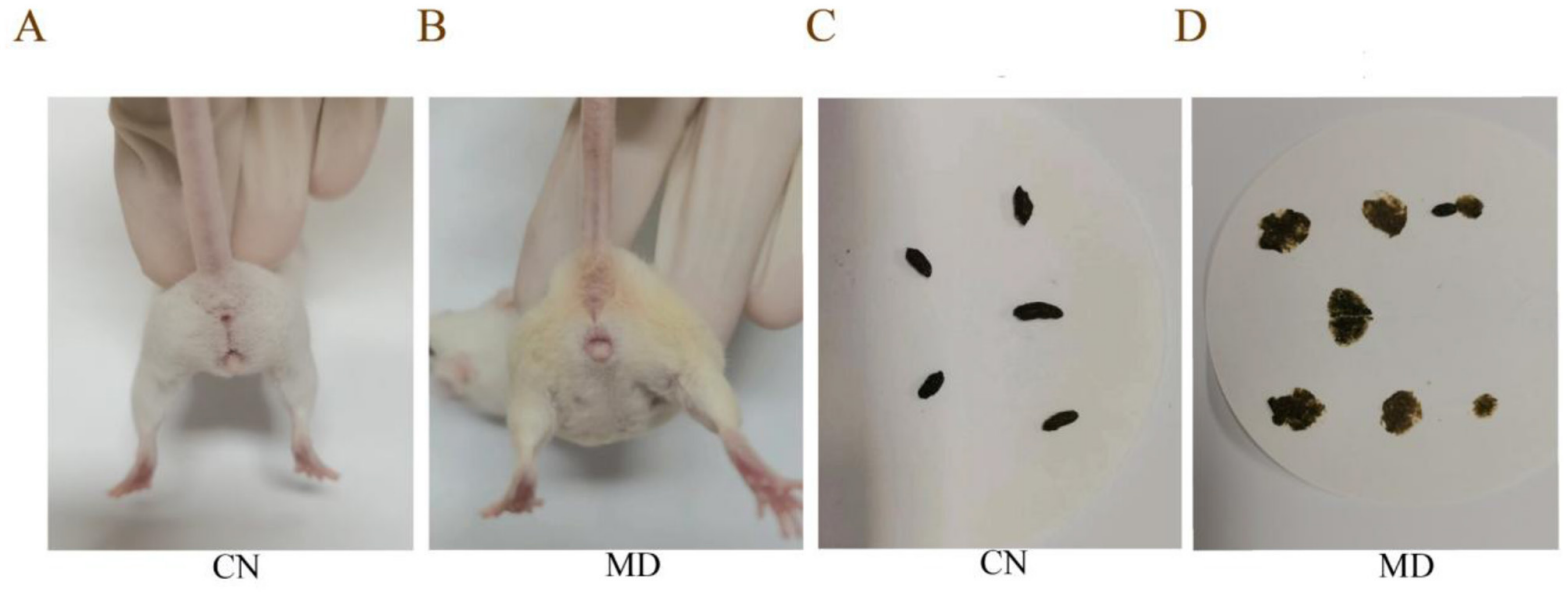
Comparison of perianal cleanliness and fecal characteristics among the different groups of mice. **(A, B)** Perianal cleanliness, **(C, D)** Fecal characteristics. The values were expressed as mean ± standard deviation. CN: Normal group (*n* = 5); MD: Model group (*n* = 5).

**Figure 2:**
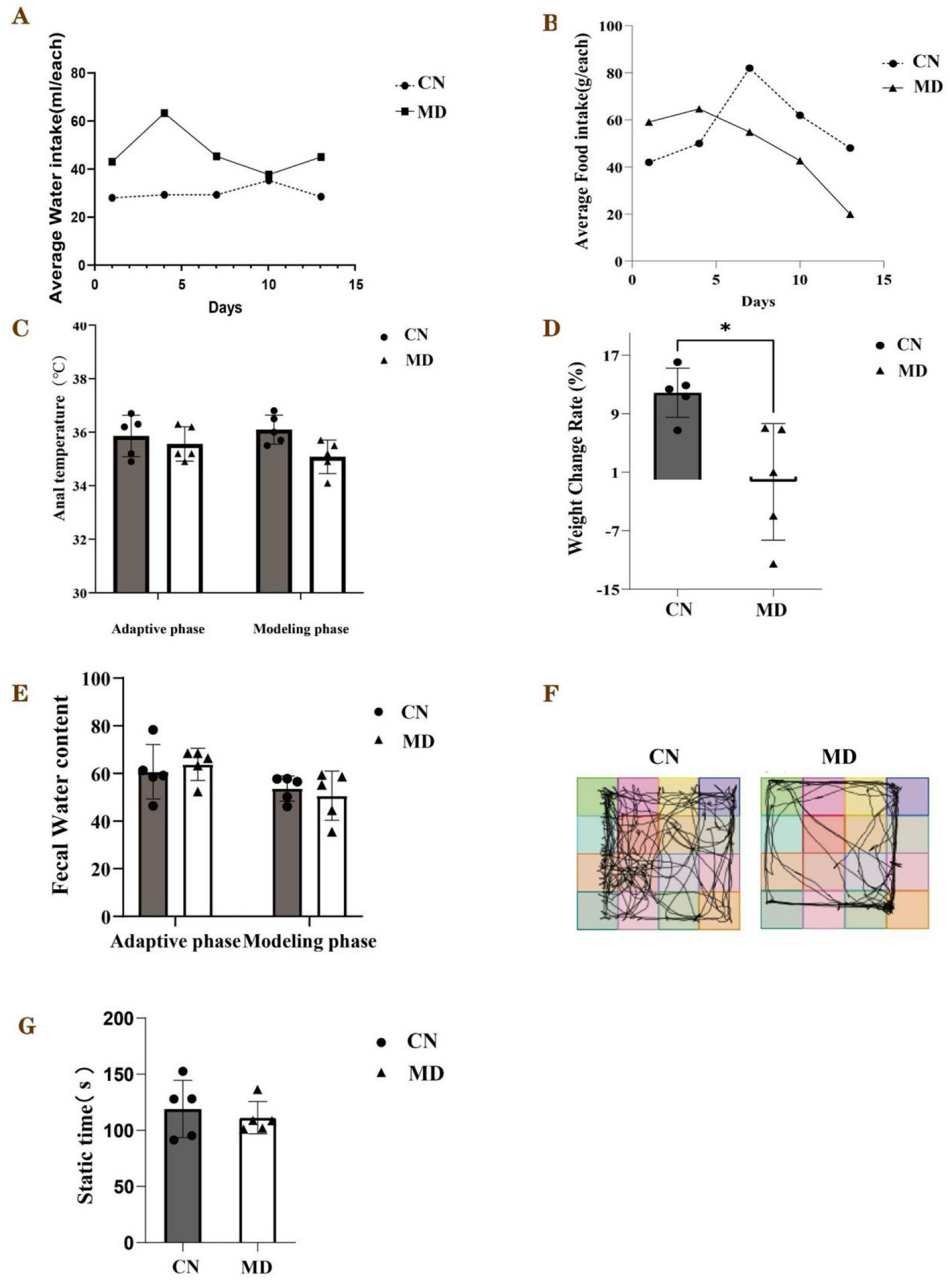
General behavioral observations and symptoms of the mice. **(A)** Average water intake, **(B)** Average food intake, **(C)** Anal temperature, **(D)** Weight change rate, **(E)** Fecal water content, **(F)** Activity tracking of mice, **(G)** The static time in each group. The values were expressed as mean ± standard deviation. *p < 0.05. CN: Normal group (*n* = 5); MD: Model group (*n* = 5).

During the modeling period, the perianal cleanliness of model group was lower than that of normal group. When exploring mouse feces characteristics, it was observed that the feces from normal group had a relatively soft yet firm consistency, and when applied to filter paper, they did not easily deform, leaving no discernible water stains on the paper. The feces of model group mice are soft, easily deformable, and stick to the tweezers when picked up (Figure 1). During the modeling period (Figure 2A), the average water intake of model group was significantly higher than that of normal group. Starting from the 8th day of modeling (Figure 2B), the average daily food intake of model group has always been lower than that of normal group. At the same time, the anal temperature and body weight are showing a downward trend (Figures 2C, D). Adenine and *Folium senna* combined with restraint tail-clamping stress method resulted in significant alterations in the overall behavior of mice and an increase in the water content of their feces (Figure 2E).

### 3.2 Comparison of the open field test results in IBS-D mice with spleen-kidney yang deficiency

The mice in normal group predominantly inhabited the central region of the testing apparatus, while they allocated minimal time to the corners and peripheral zones. In comparison to normal group, model group showed a reduced frequency of crossings between squares, fewer entries into the central square, and spent less time in the central square. These results indicate that the model mice displayed a decreased curiosity toward novel stimuli, exhibited lethargy, and had lower activity levels, which are consistent with anxiety and depression-like behaviors (Figure 2F). Furthermore, analyses of the 5-min movement distance and average speed indicated that model group's movement distance and average speed were inferior to those of normal group (Figure 2G).

### 3.3 Effects of spleen-kidney yang deficiency IBS-D on energy metabolism in mice

**Figure 3:**
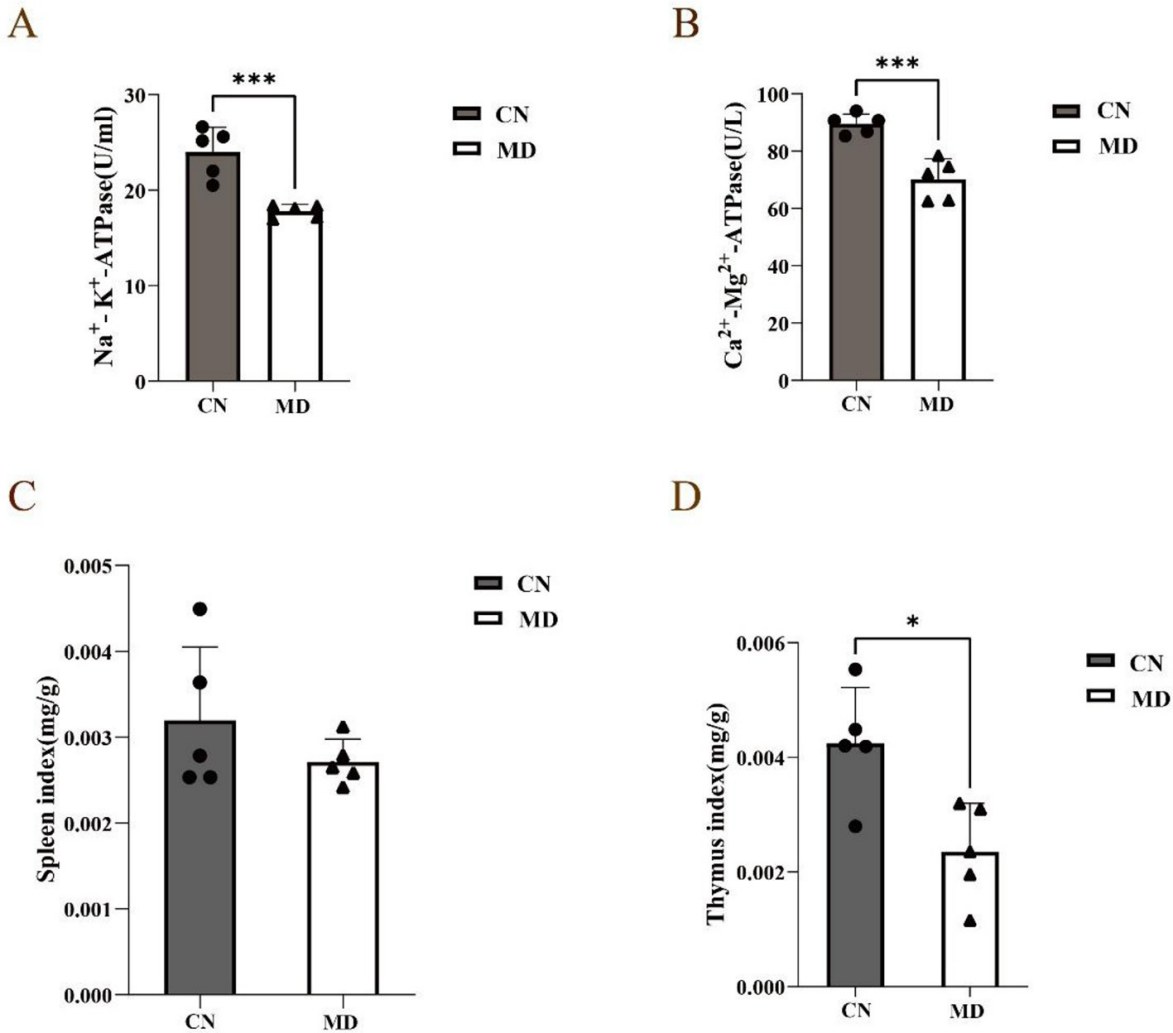
Effects of spleen-kidney yang deficiency IBS-D on serum ELISA and organ indices in mice. **(A)** Changes in Na^+^- K^+^-ATPase, **(B)** Changes in Ca^2+^-Mg^2+^-ATPase, **(C)** Spleen index, **(D)** Thymus index. The values were expressed as mean ± standard deviation. *p < 0.05. ***p < 0.001. CN: Normal group (*n* = 5); MD: Model group (*n* = 5).

Compared to normal group, the activities of Na^+^-K^+^-ATPase and Ca^2+^-Mg^2+^-ATPase in model group mice were significantly decreased (*p* < 0.001, Figure 3). This suggests that the combination of adenine and *Folium senna* combined with restraint tail-clamping stress has a certain impact on the energy metabolism of mice.

### 3.4 Effects of spleen-kidney yang deficiency IBS-D on organ indices in mice

Compared with those in normal group, the spleen and thymus indices in model group were lower (*p* < 0.05, Figures 3C, D).

### 3.5 Effects of spleen-kidney yang deficiency IBS-D on intestinal enzyme activity in mice

**Figure 4:**
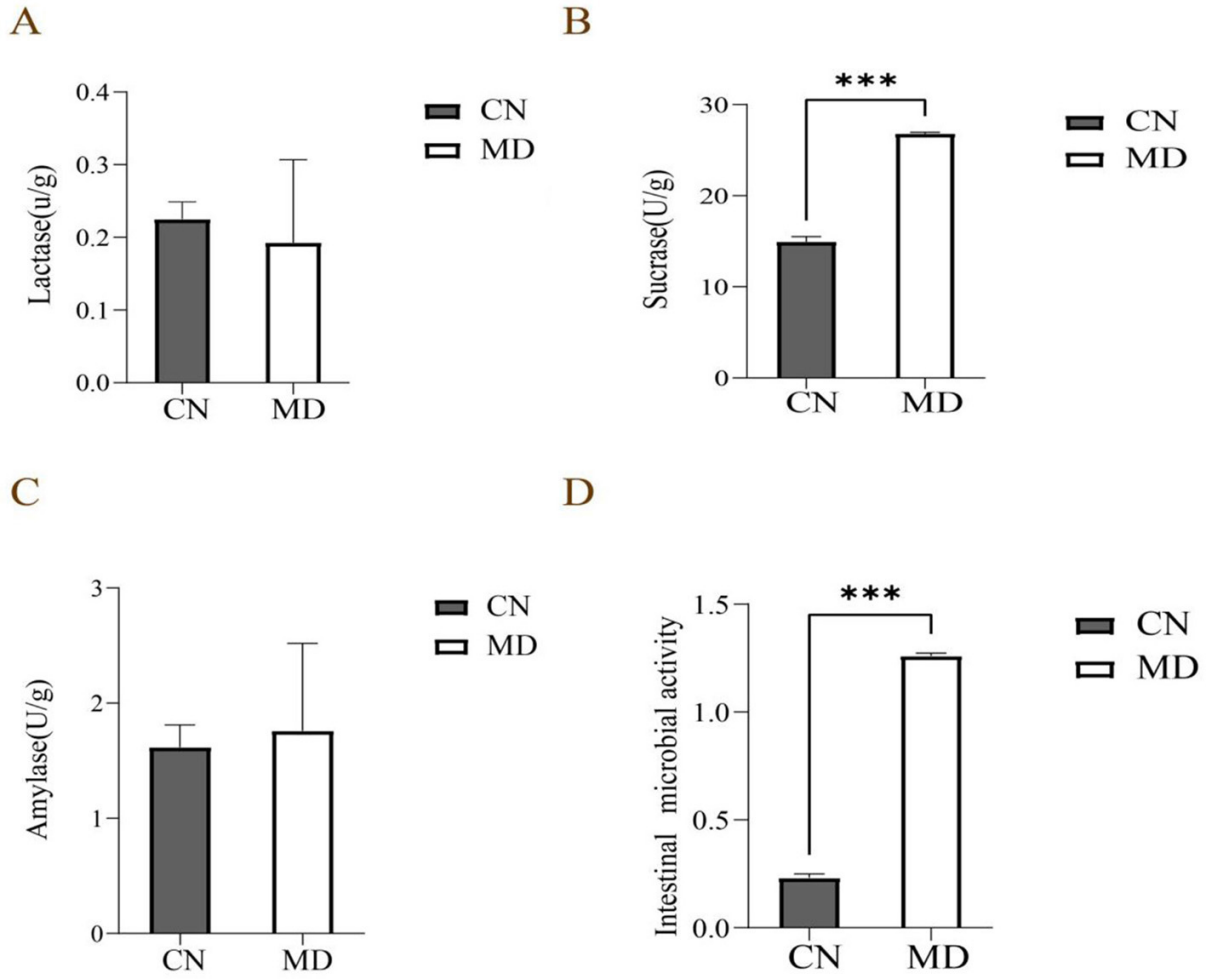
Effects of spleen-kidney yang deficiency IBS-D on intestinal digestive enzyme activity and microbial activity in mice. **(A)** Lactase activity, **(B)** Sucrase activity, **(C)** Amylase activity, **(D)** Intestinal Microbial activity. The values were expressed as mean ± standard deviation. ***p < 0.001. CN: Normal group (*n* = 5); MD: Model group (*n* = 5).

In this experiment, as shown in Figure 4, the sucrase activity in the intestinal contents of model group was significantly enhanced compared to normal group (*p* < 0.001). The amylase activity was marginally higher than that of normal group (*p* < 0.05), while the lactase activity in model group was slightly diminished relative to normal group (*p* < 0.05). The results indicate spleen-kidney yang deficiency type IBS-D model can alter the digestive enzyme activity in the intestinal contents.

### 3.6 Effects of spleen-kidney yang deficiency IBS-D on intestinal microbial activity in mice

Compared with the normal group, intestinal microbial activity in model group was significantly increased (*p* < 0.001), indicating that the model may have affected the microbial community's metabolism (Figure 4D).

### 3.7 Changes in the microbial community of the small intestinal contents

#### 3.7.1 Effects of spleen-kidney yang deficiency IBS-D on ASV count and rarefaction curves

**Figure 5:**
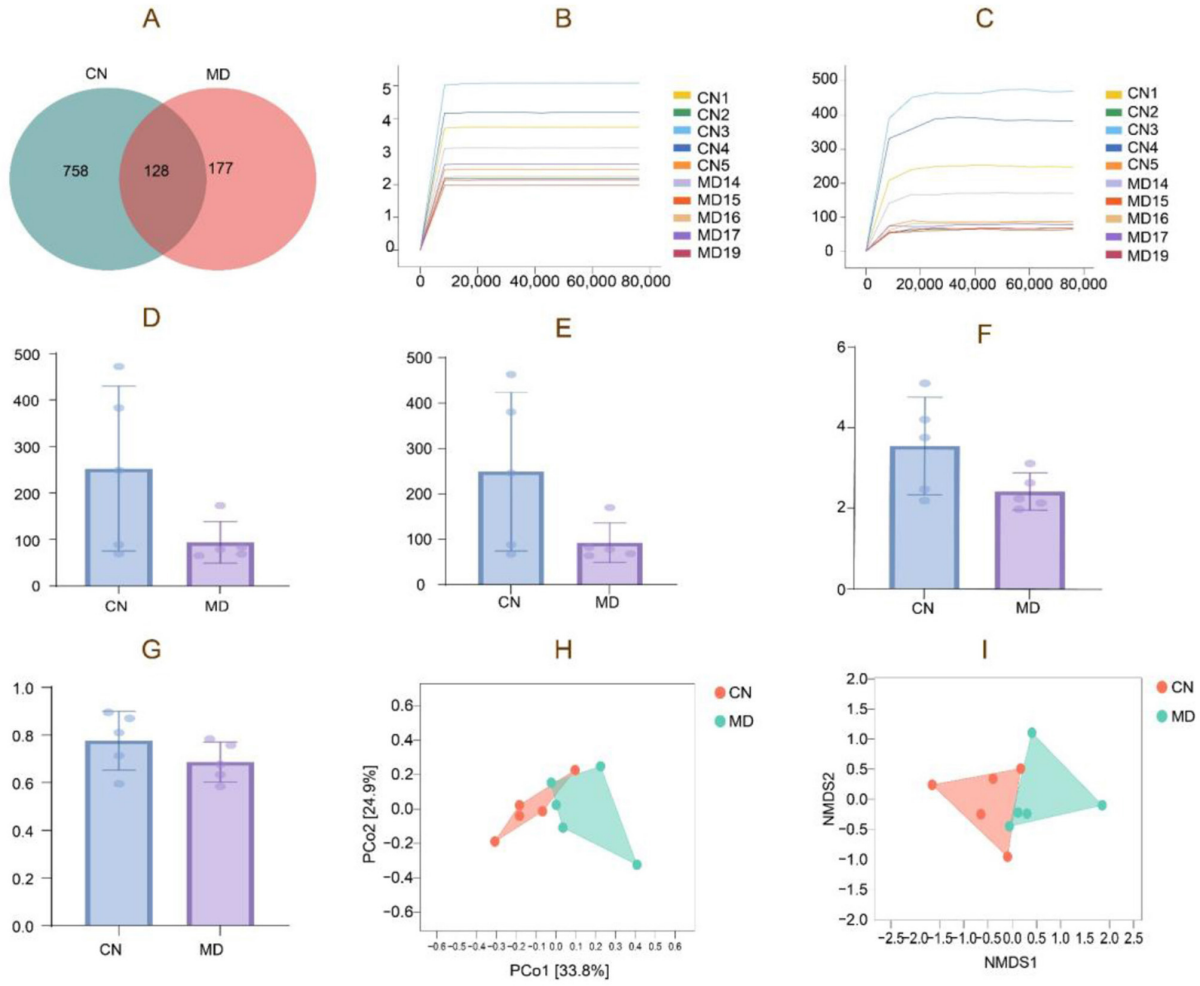
Analysis of the microbiota structure of intestinal content microbiota. **(A)** ASV quantity, **(B)** Chao1 rarefaction curve, **(C)** Shannon rarefaction curve, **(D)** Chao1 index, **(E)** Observed species index, **(F)** Shannon index, **(G)** Simpson index, **(H)** PCoA analysis. **(I)** NMDS analysis. The closer the distance between the two groups on the axes, the more similar the community composition of the two groups in the corresponding dimension. CN: Normal group (*n* = 5); MD: Model group (*n* = 5).

The Venn diagram analyzed the similarities and intersections through different sample community structures, demonstrating the commonalities and distinctiveness of samples at the ASV level. The findings of the analysis are presented in the Figure 5A. The cumulative count of common ASVs between the two groups is 128, with 758 ASVs identified in normal group and 177 ASVs in model group. The findings demonstrate that modeling decreased the quantity of ASVs in the digestive contents of mice. Chao1 and Shannon rarefaction curves exhibited an inflection point, followed by a flattening trend as the sequencing depth increased, reaching a plateau phase (Figures 5B, C).

#### 3.7.2 α diversity of the intestinal microbiota

α diversity quantifies species richness and diversity, evaluated by the Chao1, Simpson, Shannon, and Observed species indices. The α diversity data demonstrate that the diversity index of intestinal content samples has experienced a notable alteration, with model group exhibiting a reduction in Observed species, Shannon, and Simpson indices (Figures 5D–G). During the modeling process, the diversity and species richness of the intestinal microbiota exhibited a declining tendency, as indicated by the reduction in the diversity index of model group. However, these differences did not attain statistical significance (*p* > 0.05).

#### 3.7.3 β Diversity of the intestinal microbiota

β-diversity describes the variations in species composition between habitat communities, specifically the distinctions between samples. Microbial communities can be analyzed and differentiated by Non-metric Multidimensional Scaling (NMDS) and Principal Coordinates Analysis (PCoA). The samples in normal group exhibit more concentration, while the samples in model group are comparatively spread (Figure 5H). The NMDS analysis is depicted in the figure, indicating that normal group exhibits a slightly greater concentration than model group. The stress value obtained from the NMDS analysis is 0.092 (Figure 5I).

#### 3.7.4 Effects of spleen-kidney yang deficiency IBS-D on the relative abundance of intestinal contents in mice

**Figure 6:**
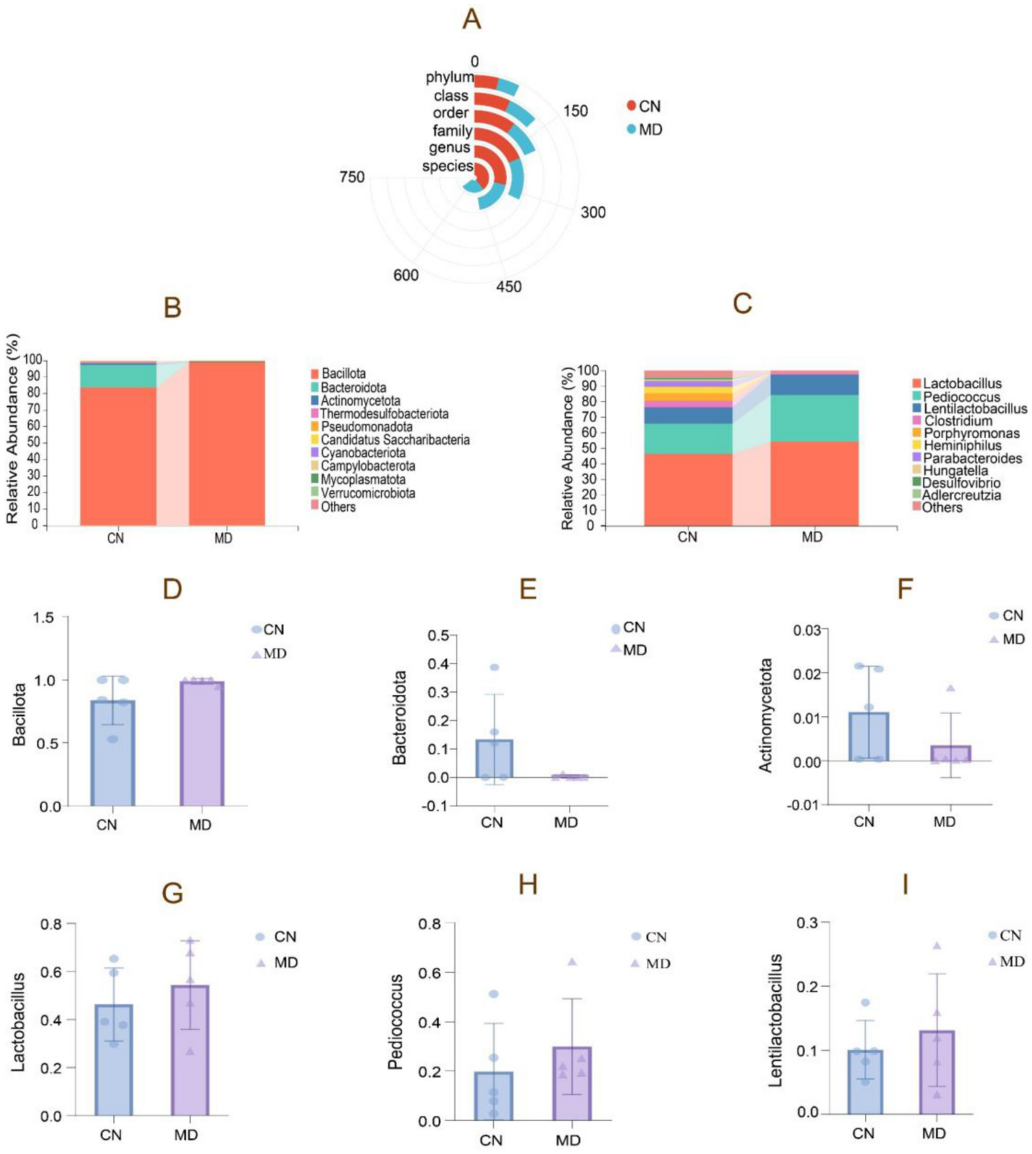
The composition of intestinal content microbiota in mice. **(A)** Radial Bar Chart, **(B)** Phylum-level relative abundance plot, **(C)** Genus-level relative abundance plot, **(D–F)** Phylum-level dominant intestinal microbiota, **(G–I)** Genus-level dominant intestinal microbiota. The values were expressed as mean ± standard deviation. CN: Normal group (*n* = 5); MD: Model group (*n* = 5).

As shown in Figure 6A, model group exhibited lower bacterial counts across all taxonomic levels compared to normal group. Figure 6B shows the relative abundance of the intestinal microbiota at the phylum level, where normal group had the highest proportion of Bacillota, followed by Bacteroidota and Actinomycetota. In contrast, model group showed an increase in Bacillota, while Bacteroidota and Actinomycetota decreased (Figures 6D–F). Figure 6C displays the relative abundance of the intestinal microbiota at the genus level. Model group showed an 8.09% increase in *Lactobacillus*, a 10.19% increase in *Pediococcus*, and a 3.05% increase in *Lentilactobacillus* compared to normal group (Figures 6G–I).

#### 3.7.5 Effects of spleen-kidney yang deficiency IBS-D on the dominant intestinal microbiota

**Figure 7:**
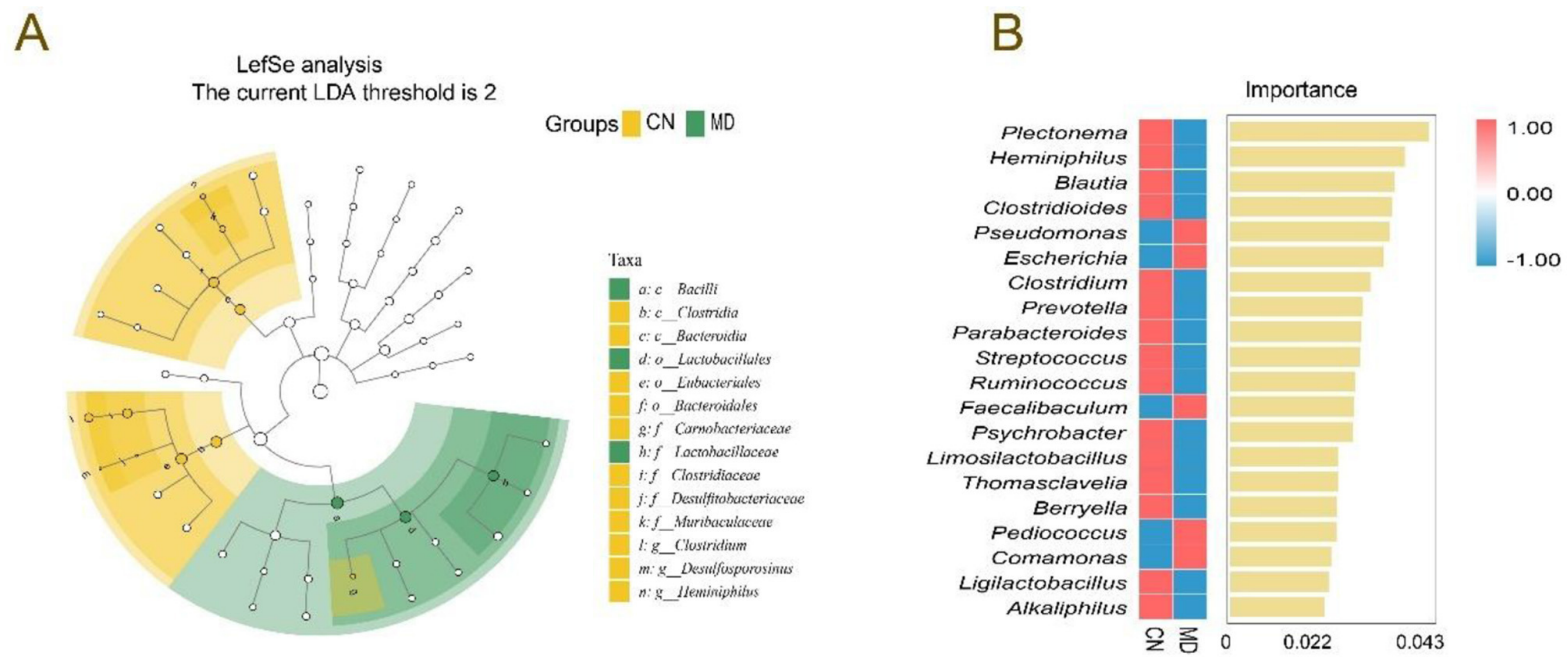
Effects of spleen-kidney yang deficiency IBS-D on the characteristic intestinal microbiota. **(A)** Evolutionary branching diagrams, **(B)** random forest diagram at the genus level. CN: Normal group (*n* = 5); MD: Model group (*n* = 5).

The study analyzed the dominant intestinal microbiota, revealing Bacillota, Bacteroidota, Actinomycetota, Thermodesulfobacteriota, and Pseudomonadota as the major phyla (Figure 7). In model group, Bacillota and Pseudomonadota increased by 15.08% and 0.43%. Respectively, compared to normal group, Bacteroidota, Actinomycetota, and Thermodesulfobacteriota decreased. A significant increase in Bacillota indicates a shift in the dominant bacterial composition. The dominant genera included *Lactobacillus, Pediococcus, Clostridium*, and *Porphyromonas*. While the genus-level bacterial composition shifted, this change was not statistically significant.

#### 3.7.6 Effects of spleen-kidney yang deficiency IBS-D on characteristic intestinal microbiota

LEfSe (LDA score > 3) was used to identify significant microbial taxa, and we aimed to explore the differences in the intestinal microbiota between normal group and model group (Figure 7A). In normal group, the dominant bacteria included *Clostridium, Desulfosporosinus* and *Heminiphilus*, no characteristic bacteria were identified in MD group. In the random forest plot, the top 20 most abundant bacterial genera were selected for analysis. *Plectonema, Heminiphilus, Blautia*, and *Clostridioides* were the dominant genera in normal group, while *Pseudomonas* and *Escherichia* were the dominant genera in model group (Figure 7B).

#### 3.7.7 Effects of spleen-kidney yang deficiency IBS-D on the functional analysis of the intestinal microbiota in mice

**Figure 8:**
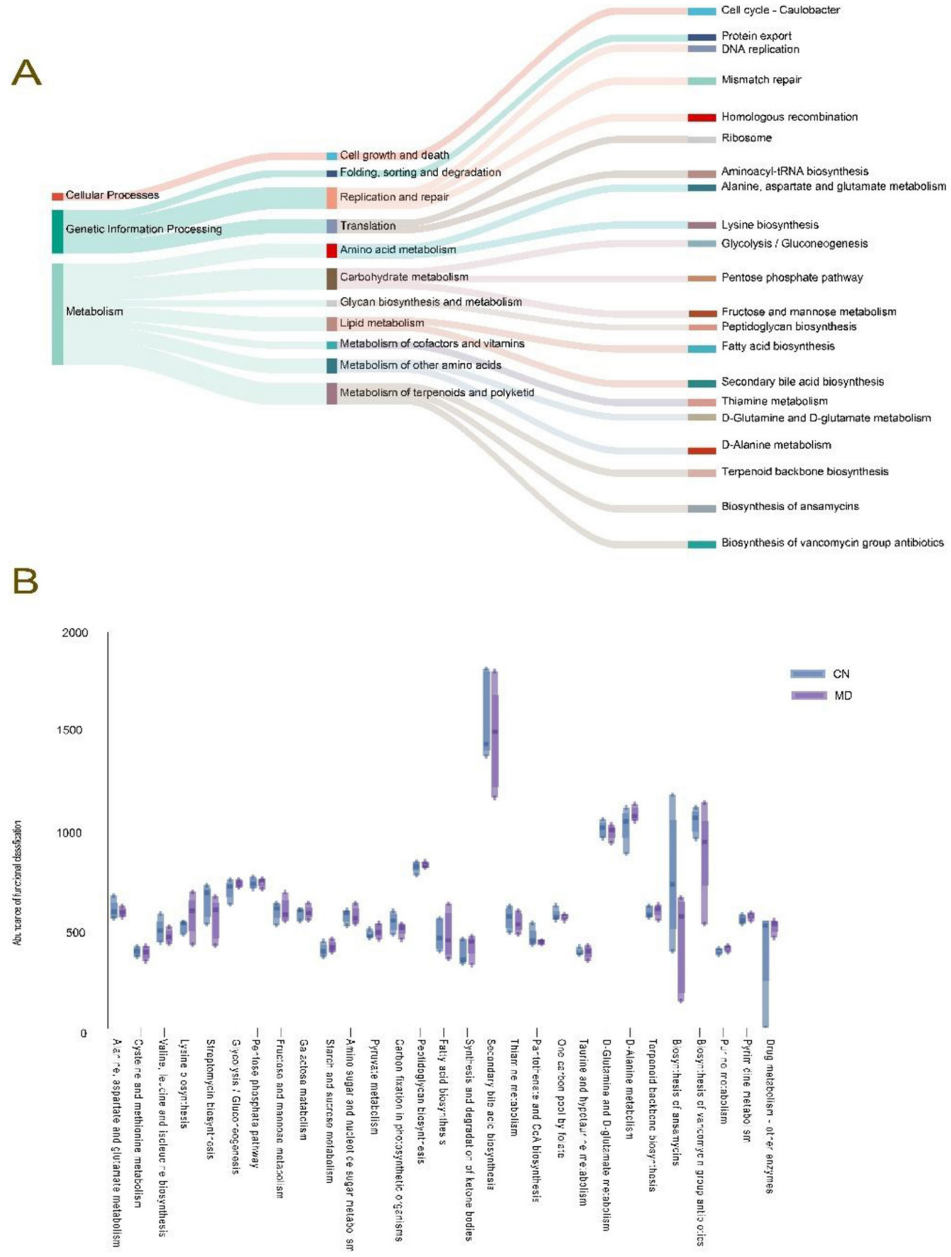
Effects of spleen-kidney yang deficiency IBS-D on the functional analysis of the intestinal microbiota in mice. **(A)** Tracking Sankey plots, **(B)** Metabolic function intergroup comparative box line plots (Level 3). The values are expressed as the means ± standard deviations. CN: Normal group (*n* = 5); MD: Model group (*n* = 5).

To determine the metabolic and functional changes in the intestinal microbiota of mice, we categorized the intestinal microbiota. The functional analysis revealed that the intestinal microbiota could be divided into three major functional categories (Figure 8A), with the double median of 39 subcategories being > 380.4145. Among the top 29 KEGG pathways analyzed, metabolic functions accounted for 61.70% of the genes. The main impacts were observed on amino acid metabolism, energy metabolism, carbohydrate metabolism, lipid metabolism, and nucleotide metabolism. Although no significant changes were noted, compared with normal group, model group presented a decrease in the metabolic function of the secondary bile acid biosynthesis pathway (Figure 8B).

#### 3.7.8 Correlation analysis of the intestinal microbiota

**Figure 9:**
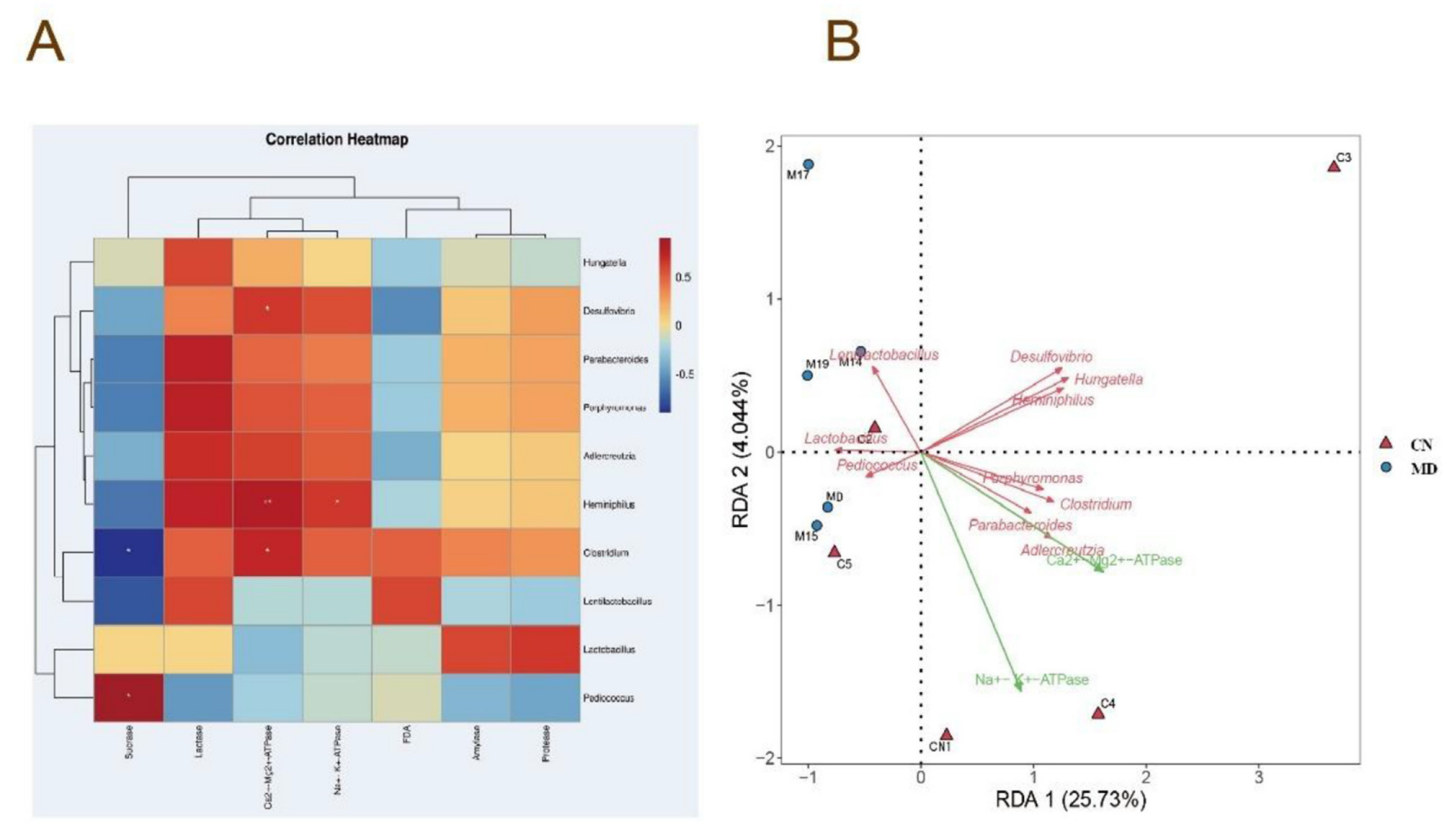
Correlation analysis of the intestinal microbiota. **(A)** Heatmap depicting the correlation between intestinal enzyme activities in mice and microbial activity (FDA), Na^+^-K^+^-ATPase activity, and Ca^2^^+^-Mg^2^^+^-ATPase activity. **(B)** RDA redundancy analysis. CN: Normal group (*n* = 5); MD: Model group (*n* = 5).

To explore the relationships among energy metabolism, digestive enzyme activity in the intestinal contents, microbial activity, and the microbiota in mice with spleen-kidney yang deficiency IBS-D, Spearman correlation analysis was conducted. *Heminiphilus, Clostridium*, and *Desulfovibrio* were significantly positively correlated with Ca^2+^-Mg^2+^-ATPase. *Heminiphilus* also presented a significant positive correlation with Na^+^-K^+^-ATPase, while *Pediococcus* presented a significant positive correlation with sucrase, and *Clostridium* presented a significant negative correlation with sucrase (Figure 9A). The RDA results indicated that *Lactobacillus* and *Lentilactobacillus* were negatively correlated with Na^+^-K^+^-ATPase and Ca^2+^-Mg^2+^-ATPas. In contrast, *Pediococcus, Clostridium, Porphyromonas, Heminiphilus, Parabacteroides, Hungatella, Desulfovibrio*, and *Adlercreutzia* were positively correlated with both Na^+^-K^+^-ATPase and Ca^2+^-Mg^2+^-ATPase (Figure 9B).

## 4 Discussion

### 4.1 The relationship between spleen-kidney yang deficiency IBS-D and ATPase

The pathogenesis of IBS-D is complex and multifactorial. Liver qi stagnation, spleen deficiency, and impaired qi flow are considered to be critical factors contributing to the onset and progression of IBS-D (Liu et al., 2020). In light of these findings, this study replicates previous research conducted by our team, using adenine and *Folium senna* combined with restraint tail-clamping stress to establish a spleen-kidney yang deficiency IBS-D model. From the perspective of TCM, the functions of kidney yang qi, spleen transportation, and mitochondrial energy metabolism are similar, as they all play a crucial role in maintaining normal physiological functions (Qi et al., 2018). When kidney-yang is insufficient, the body experiences functional decline and metabolic slowdown, which is similar to the energy deficiency caused by mitochondrial dysfunction in modern medicine (Luo et al., 2022). In cases of spleen deficiency, the number of mitochondria decreases, their structure becomes abnormal, and the activity of related enzymes (such as ATPase, superoxide dismutase, and respiratory chain complex I) is reduced, leading to a decrease in ATP content (Wang et al., 2019). Both spleen and kidney Yang deficiency result in abnormal energy metabolism, affecting the body's ability to supply sufficient energy. Some studies suggest that deficiencies in spleen-kidney yang lead to insufficient generation of qi and blood, resulting in a shortage of energy substrates (such as glucose, fatty acids, amino acids, vitamins, etc.) required for mitochondrial energy metabolism (Zhang and Song, 2020).

The activity levels of Na^+^-K^+^-ATPase and Ca^2^^+^-Mg^2^^+^-ATPase are key indicators of cellular energy metabolism and are closely associated with mitochondrial function (Tremellen and Pearce, 2012). These enzymes play a pivotal role in maintaining cellular energy balance, and their activity levels can reflect the overall metabolic state of the organism. These metabolic disturbances can impair mitochondrial enzyme function, disrupt metabolic regulation, and ultimately lead to energy deficiencies and the development of related diseases. This impacts mitochondrial enzyme function and metabolic regulation, resulting in metabolic disorders and inadequate energy production. Prior research indicates that in models of spleen insufficiency, the activities of mitochondrial superoxide dismutase and glutathione peroxidase are diminished, but the activities of Na^+^-K^+^-ATPase and Ca^2^^+^-Mg^2^^+^-ATPase are markedly reduced, accompanied with lipid peroxidation damage and disruptions in energy metabolism (Hu et al., 2017). Likewise, Qiu Lin observed a reduction in Na^+^-K^+^-ATPase and ATP levels in rat models exhibiting renal yang deficiency, resulting in disturbances in energy metabolism (Qiu et al., 2018). This study's results demonstrate that in the spleen-kidney yang deficiency IBS-D mice model, the activities of Na^+^-K^+^-ATPase and Ca^2^^+^-Mg^2^^+^-ATPase are diminished, corroborating the aforementioned findings.

### 4.2 Intervention in the spleen-kidney yang deficiency IBS-D model induces alterations in the intestinal microbiota and intestinal contents in mice

The intestinal microbiota plays an important role in influencing the physiological functions of the host, and therefore, some scholars refer to the intestinal microbiota as the “functional organ” of the host (Correale et al., 2022). Yang deficiency is closely related to the occurrence of intestinal, metabolic, and immune system diseases, which aligns with digestive disorders and immune dysfunction caused by intestinal dysbiosis (Dai et al., 2025). When spleen and stomach yang is deficient, the transformation of food is impaired, leading to intestinal microbiota imbalance. If kidney-yang is insufficient, the production of qi and blood is deficient, and the intestines lack proper nourishment, also causing microbiota disruption. On the other hand, intestinal dysbiosis interferes with the spleen and stomach's function, damages vital qi, weakens immune function, and increases cold-damp accumulation, thus exacerbating Yang deficiency symptoms and creating a vicious cycle (Ma et al., 2021). Thus, intestinal microbiota is closely related to spleen-kidney yang deficiency IBS-D.

Sequencing analysis of small intestinal contents revealed that spleen-kidney yang deficiency IBS-D significantly altered the microbial community structure. In this study, compared with the normal group, the model group showed a significant reduction in the number of associated ASVs in the intestinal microbiota. Although α-diversity was reduced, this difference did not reach statistical significance, possibly due to the small sample size and individual variations in the mice. β-diversity analysis revealed changes in the overall structure of the intestinal microbiota. Analysis of the dominant intestinal microbiota showed an increased relative abundance of Bacillota and Pseudomonadota at the phylum level, and *Lactobacillus, Pediococcus*, and *Lentilactobacillus* at the genus level. These results suggest that spleen-kidney yang deficiency IBS-D alters the microbial community structure of intestinal contents.

### 4.3 Correlation between intestinal microbiota and ATPase activity in spleen-kidney yang deficiency IBS-D

The intestinal microbiota contributes to host energy metabolism by generating metabolites such butyrate, propionate, and acetate, and by metabolizing complex food polysaccharides and host glycans to supply energy (Heiss and Olofsson, 2018). The intestinal microbiome also affects mitochondrial activity in intestinal epithelial cells via its metabolites, hence influencing energy metabolism (Nogal et al., 2021). Mitochondria in intestinal epithelial cells govern energy metabolism by adjusting redox state, suppressing immunological responses, preserving intestinal barrier integrity, and regulating pathogen activity, ultimately driving ATP synthesis, hence affecting the composition and function of the intestinal microbiota (Zhang et al., 2018; Zheng, 2020).

Our findings indicate substantial positive connections between *Heminiphilus, Clostridium, Desulfovibrio*, and Ca^2+^-Mg^2+^-ATPase activity in mice with spleen-kidney yang deficiency IBS-D, along with a notable positive correlation between *Heminiphilus* and Na^+^-K^+^-ATPase activity. *Heminiphilus*, a constituent of the Bacteroidetes phylum, is frequently located in the human upper respiratory system and intestine. The direct correlation between *Heminiphilus* and IBS-D is ambiguous; nevertheless, it may indirectly influence intestinal immunological or inflammatory responses within the intestinal microbiota. Additional inquiry into its function in the gastrointestinal tract is required (Park et al., 2021). *Clostridium* modulates intestinal microbiota composition and immunological responses via its metabolites. It metabolizes diverse substances, such as carbs, proteins, and organic acids, to generate SCFAs, hence improving host energy assimilation (Du et al., 2024). *C. butyricum*, a probiotic, synthesizes butyrate, which confers advantageous benefits by enhancing tight junction proteins, mucins, and anti-inflammatory cytokines, while suppressing pro-inflammatory cytokines, modifying intestinal microbiota composition, and diminishing oxidative stress (Qian et al., 2025). *Desulfovibrio*, regarded as a detrimental bacterium, generates hydrogen sulfide in the gastrointestinal tract, which is toxic to the intestinal epithelium and may lead to gastrointestinal disorders (Crispim et al., 2018). Despite being contentious, considerable evidence indicates that *Desulfovibrio* is linked to inflammatory situations (Taglialegna, 2024). In summary, *Heminiphilus, Clostridium*, and *Desulfovibrio* are associated with ATPase activity in mice exhibiting spleen-kidney yang deficiency IBS-D.

Based on current findings, future clinical diagnosis of spleen-kidney yang deficiency IBS-D should integrate both TCM syndrome differentiation and objective diagnostic indicators (such as ATPase levels and the abundance of specific bacterial genera). In terms of treatment, modulating the microbiota may help restore energy metabolism and enhance therapeutic effects. Future research should focus on validating these treatment targets in clinical cohorts to promote personalized treatment strategies for IBS-D patients with spleen-kidney yang deficiency.

## 5 Conclusion

Reduced Na^+^-K^+^-ATPase and Ca^2^^+^-Mg^2^^+^-ATPase activities, increased sucrase and amylase activities, decreased lactase activity, and enhanced intestinal microbial activity may constitute key mechanisms underlying the development of spleen-kidney yang deficiency IBS-D. This study preliminarily suggests that the “microbiota-ATPase-energy metabolism” axis may be disrupted in mice with spleen-kidney yang deficiency IBS-D, highlighting its potential critical role in the pathological state. However, further investigation using more systematic and refined experimental designs is required to deepen our understanding of this mechanism.

## Data Availability

The original contributions presented in the study are publicly available. This data can be found in here: https://www.ncbi.nlm.nih.gov, number PRJNA1177239.
