# Correction: One mechanism of spleen-kidney yang deficiency IBS-D: intestinal microbiota affect ATPase

**DOI:** 10.3389/fmicb.2026.1919180

**Published:** 2026-07-14

**Authors:** Qi Long, Liwen Li, Na Deng, Zhoujin Tan

**Affiliations:** 1 School of Traditional Chinese Medicine, Hunan University of Chinese Medicine, Changsha, China; 2 Hunan Key Laboratory of Traditional Chinese Medicine Prescription and Syndromes Translational Medicine, Changsha, China

**Keywords:** intestinal microbiota, ATPase, spleen-kidney yang deficiency IBS-D, energy metabolism, intestinal digestive enzymes

In the published article, there were two errors in **Materials and methods**, section *2.1 Materials*, subsection *2.2.1 Grouping and modeling methods*, Paragraph 1. The duration of *Folium senna* administration was incorrectly stated as “7 days” and should be corrected to “5 days.” The duration of adaptive feeding was incorrectly stated as “7 days” and should be corrected to “3 days.

The original passage is as follows: “After 7 days of adaptive feeding, 20 female mice were randomly assigned to normal and model group with 10 mice in each group. The IBS-D mouse model with spleen-kidney yang deficiency was induced by the administration of adenine and *Folium sennae* decoction combined with restraint-tail clamping stress on the basis of a previous study from our research team. In the week prior to modeling, the model group was gavaged with an adenine suspension (50 mg/(kg·d), 0.4 mL per mouse, once a day, for 14 consecutive days). Starting from the 8th day of modeling, the limbs of model group mice were restrained via a centrifuge tube, and the distal 1/3 of their tail was clamped with a long tail clamp. Both the restraint and intermittent tail-clamping durations were 1 h, which lasted for 7 consecutive days. In the afternoon, model group mice were gavaged with *Folium sennae* decoction (10 g/(kg·d), 0.4 mL/mouse, once a day, for 7 consecutive days), whereas normal group received sterile water at the same frequency and volume (Deng et al.,2024).”

The corrected text reads: “After 3 days of adaptive feeding, 20 female mice were randomly assigned to normal and model group with 10 mice in each group. The IBS-D mouse model with spleen-kidney yang deficiency was induced by the administration of adenine and *Folium sennae* decoction combined with restraint-tail clamping stress on the basis of a previous study from our research team. In the week prior to modeling, the model group was gavaged with an adenine suspension (50 mg/(kg·d), 0.4 mL per mouse, once a day, for 14 consecutive days). Starting from the 8th day of modeling, the limbs of model group mice were restrained via a centrifuge tube, and the distal 1/3 of their tail was clamped with a long tail clamp. Both the restraint and intermittent tail-clamping durations were 1 h, which lasted for 7 consecutive days. Starting from day 10, model group mice were gavaged with *Folium sennae* decoction in the afternoon (10 g/(kg·d), 0.4 mL/mouse, once a day, for 5 consecutive days), whereas normal group received sterile water at the same frequency and volume (Deng et al., 2024).”

The original version of this article has been updated.

